# Research Hotspots and Trends Analysis of TFEB: A Bibliometric and Scientometric Analysis

**DOI:** 10.3389/fnmol.2022.854954

**Published:** 2022-04-21

**Authors:** Runjin Zhou, Xiaoling Lin, Dongmin Liu, Zhao Li, Jingchun Zeng, Xingdong Lin, Xiaodi Liang

**Affiliations:** ^1^Medical College of Acupuncture-Moxibustion and Rehabilitation, Guangzhou University of Chinese Medicine, Guangzhou, China; ^2^The First Clinical Medical College of Guangzhou University of Chinese Medicine, Guangzhou, China; ^3^The First Affiliated Hospital of Guangzhou University of Chinese Medicine, Guangzhou, China; ^4^The Third Affiliated Hospital of Guangzhou University of Chinese Medicine, Guangzhou, China

**Keywords:** transcription factor EB, CiteSpace, VOSviewer, scientometric analysis, WoSCC

## Abstract

**Objective:**

To explore the development context, research hotspots and frontiers of Transcription factor EB (TFEB) from 1991 to 2021 by bibliometric analysis.

**Methods:**

Publications about TFEB research from 1991 to 2021 were retrieved from the Web of Science Core Collection (WoSCC). Excel 2007 was used to collect basic information, including publications, research areas. VOSviewer 1.6.17 was used to analyze co-authorship of countries, institutes and authors. Co-citation of cited authors, cited references were analyzed by CiteSpace V.5.8.R3. In addition, CiteSpace was used to analyze keywords cluster and forecast research frontiers.

**Results:**

A total of 1,059 literatures were retrieved, including 1,340 research institutes and 393 academic journals. The main area of research related to TFEB is biology (340), the most published country and institutes were the United States (487) and Baylor College of Medicine (70). Settembre C owned the highest co-citations (663). Trending keywords may indicate frontier topics, including “Alzheimer’s disease,” “Parkinson’s disease,” “(p21; q12),” “melanoma,” “pancreatic cancer,” “breast cancer,” “calcineurin,” “TFE3,” “trehalose,” and “curcumin.”

**Conclusion:**

This research provides valuable information for the study of TFEB. Disease research focuses more on neurodegenerative diseases (NDs) and tumors. Trehalose and curcumin are novel agents acting on TFEB. Rap-TRPML1-Calcineurin-TFEB and TFE3 are increasing signal pathway researches, similarly, the molecular biological mechanism of TFEB needs further exploration.

## Introduction

Transcription factor EB (TFEB), as one of the main factors in the microphthalmia-associated transcription factor (MiTF) family. Like other transcription factors MiTF, TFE3, and TFEC, it has a basic helix-loop-helix leucine zipper (bHLH-ZiP) domain which is highly similar to its dimerization. The bHLH-ZiP domain mediates MiT family members to form homodimers or heterodimers, and then participate in the activation of target gene transcription (Steingrímsson et al., 2004).

Autophagy-lysosomal pathway (ALP) is involved in the degradation of long-lived proteins. Functional disorder of ALP leads to protein aggregation, resulting in accumulation of abnormal proteins and ineffective organelles, such as Alzheimer’s disease (AD) caused by amyloid β-protein (Aβ) deposition (Reddy and Oliver, 2019) and alpha-synuclein accumulation result in Parkinson’s disease (PD) (Cerri and Blandini, 2019). In recent years, It has been found that TFEB, as the main regulator of ALP, combines with E-box, M-box promoter or coordinate lysosomal expression and regulation (CLEAR) to regulate gene expression of lysosomal (Sardiello et al., 2009), participates in autophagosome formation, autophagosome-lysosome fusion and degrade autophagy substrates (Settembre et al., 2011). In the state of starvation or low-sugar, Ca^2+^ is released from lysosomal via mucolipin 1 (MCOLN1), causing microdomains of high Ca^2+^ concentration near the surface of lysosomes (Medina et al., 2015), which activates calcineurin (CN) and dephosphorylates TFEB, so that Lysosomal exocytosis gets strengthened for enhancing clearance within the cell (Medina et al., 2011). Animal experiments indicate that TFEB has great potential in improving neurodegenerative diseases (NDs) (Xiao et al., 2015; Zheng et al., 2021), tumors (Liang et al., 2018; Li et al., 2020) and other diseases, providing a new direction for the clinical treatment and prevention of various human diseases.

In recent years, there has been increasingly researches on the autophagy pathway, and numerous papers on TFEB research have been published. However, there is currently a lack of systematic analysis on the development trend of this domain. Information visualization technology is a valuable research method and means emerged in the fields of scientometrics and knowledge metrology in recent years. Bibliometric visualization analysis is a quantitative analysis combining mathematics and statistical methods, which can intuitively highlight the metrological characteristics of research literature in a certain field, and help researchers grasp the development characteristics of the field over time (Ellegaard and Wallin, 2015). Therefore, this study uses bibliometric analysis to systematically review the TFEB research from 1991 to 2021. We aim to understand the co-citation of the literature, build a research cooperation network, and evaluate research trends and frontiers.

## Materials and Methods

### Data Source and Search Strategy

Literature retrieval was done online through the Science Citation Index-Expanded (SCI-E) of the Web of Science Core Collection (WoSCC) on February 23, 2022. The data retrieval strategy was: topic: (Transcription Factor EB) OR topic: (Transcription Factors EB) OR topic: (Transcript* Factor* EB) OR topic: (TFEB) OR topic: (Tfeb), index = SCI-EXPANDED, time span: 1991-01-01 to 2021-12-31. The following search string was used: document type: (Article), language = English.

### Data Collection

**FIGURE 1:**
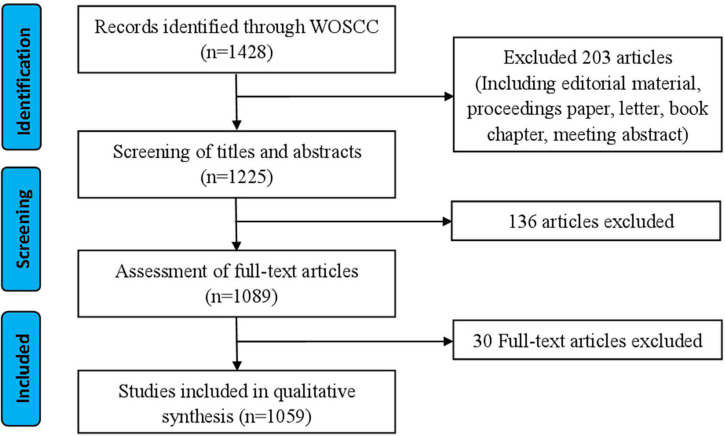
Study flow diagram.

Raw data from WoSCC was downloaded and verified by RZ and XLL, respectively, flow chart of research inclusion in Figure 1. The data were then imported into VOSviewer 1.6.17 (Leiden University, Van Eck NJ) and CiteSpace V.5.5.R2 (Drexel University, Philadelphia, PA, United States), the information generated by the software is imported into Excel 2007 (Redmond, WA, United States).

### Statistical Methods

**FIGURE 2:**
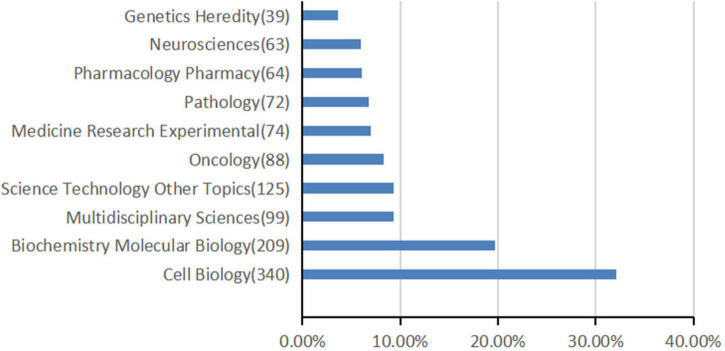
The top 10 most frequently appearing research areas in TFEB research.

Web of Science Core Collection database was used to analyze the characteristics of the literature, including countries/region, institutes, authors, journal sources, research fields, number of citations, number of annual publications, and impact factor. Excel 2007 was used to analyze the publications and citations trend (Figure 2). In this figure, the variable x represents the year, and y represents the number of publications and citations.

VOSviewer is a bibliometric analysis software jointly developed by Nees Jan van Eck and Ludo Waltman for mapping scientific knowledge (van Eck and Waltman, 2010). It has great advantages in cluster analysis, using for the co-occurrence of keywords and collaboration networks of countries/institutes/authors There were three types of mapping generated: Network Visualization, Overlay Visualization, and Density Visualization. For the Overlay Visualization, it generated a timeline in the lower right corner of the image, where blue indicated earlier publication years and darker yellow indicated more recent research.

CiteSpace is a tool for visualizing and analyzing trends and patterns in scientific papers (Chen, 2004, 2006). It was used for reference/author co-citation analysis to construct a co-citation map. parameters were as follows: time slice (1991–2021), years per slice (1), term source (all selected), node type (one at a time), selection criteria (50), pruning (Pathfinder, pruning sliced networks, pruning the merged network), and visualization (cluster view-static, show merged network). We define that the nodes in the co-citation knowledge graph represent different documents, and the size of the node is proportional to the number of references cited in a specific period. This will make it more intuitive to observe the trend of various research hotspots over time. In addition, we analyzed the characteristics related to reference citation clusters, in which the purple reference ring represented the research’s high mediating centrality, which played a role in connecting various citations, and the orange represented the newly emerging research (Chen et al., 2012).

## Results

### Annual Publications and Growth Forecast

A total of 1,059 literatures were included from 1991 (*n* = 2) to 2021 (*n* = 203), the citations of these literatures also increased dramatically from 1996 (*n* = 9) to 2021 (*n* = 2,855), with a total of 16,338 citations (Supplementary Figure 1).

### Research Areas Analysis

A total of 80 research areas were represented, cell biology (*n* = 340), biochemistry molecular biology (*n* = 209), and multidisciplinary sciences (*n* = 99) occupied the main position. Figure 2 shows the top 10 research areas in TFEB from 1991 to 2021.

### Distribution of Journals and Cited Articles

A total of 393 academic journals have published publications on TFEB. Table 1 lists the top 10 journals with a total of 256 articles, accounting for 24.17% of the total number of articles. *Autophagy* published the most articles (77 articles, 7.27%), followed by Journal of *Nature Communication* (30 articles, 2.83%), *Cell death & disease* (27 articles, 2.55%). Supplementary Figure 2 displayed the dual-map overlay of journals (Chen and Leydesdorff, 2014), the left and right sides corresponded to the citation map and the cited journal map, respectively. These labels represented the disciplines covered by the journal. Lines on the map start from the left and end on the right, representing citation links. There was one citation path, the yellow path, publications in molecular/biology/immunology journals mostly cited journals in molecular/biology/genetics area. Table 2 shows the 10 most frequently cited articles.

**TABLE 1:** The top 10 journals that published articles in TFEB research.

Rank	Journal	Country	Count	Percent	IF 2021
1	Autophagy	United States	77	7.27	16.016
2	Nature Communications	England	30	2.83	14.919
3	Cell Death & Disease	England	27	2.55	8.469
4	Journal of Biological Chemistry	United States	24	2.27	5.157
5	Scientific Reports	England	23	2.17	4.379
6	American Journal of Surgical Pathology	United States	18	1.70	6.394
7	International Journal of Molecular Sciences	United States	17	1.61	5.923
8	Proceedings of the National Academy of Sciences of the United States of America	United States	16	1.51	11.205
9	Biochemical and Biophysical Research Communications	United States	12	1.13	3.575
10	Embo Journal	United States	12	1.13	11.598

**TABLE 2:** Top 10 most cited articles on TFEB.

Rank	References	Title	Journal	Cited
1	Settembre et al., 2011	TFEB Links Autophagy to Lysosomal Biogenesis	Science	1,649
2	Sardiello et al., 2009	A Gene Network Regulating Lysosomal Biogenesis and Function	Science	1,315
3	Settembre et al., 2012	A lysosome-to-nucleus signaling mechanism senses and regulates the lysosome via mTOR and TFEB	Embo Journal	1,042
4	Roczniak-Ferguson et al., 2012	The Transcription Factor TFEB Links mTORC1 Signaling to Transcriptional Control of Lysosome Homeostasis	Sci Signal	692
5	Martina et al., 2012	MTORC1 functions as a transcriptional regulator of autophagy by preventing nuclear transport of TFEB	Autophagy	625
6	Medina et al., 2015	Lysosomal calcium signaling regulates autophagy through calcineurin and TFEB	Nature Cell Biology	607
7	Settembre et al., 2013a	TFEB controls cellular lipid metabolism through a starvation-induced autoregulatory loop	Nature Cell Biology	526
8		Pathogenic Lysosomal Depletion in Parkinson’s Disease	Journal of Neuroscience	493
9		Characterization of the CLEAR network reveals an integrated control of cellular clearance pathways	Human Molecular Genetics	488
10	Perera et al., 2015	Transcriptional control of autophagy-lysosome function drives pancreatic cancer metabolism	Nature	426

### Distribution of Countries and Institutes

A total of 55 countries/regions have published research publications on TFEB, extensive cooperation between countries/regions has been observed (Supplementary Figure 3). Table 3 lists the top 10 countries/regions in the number of publications, of which the United States is the most, followed by Mainland China, Italy, Japan, and Canada.

**TABLE 3:** Top 10 countries and institutions in the number of publications.

Rank	Country/Region	Count	Institute	Count
1	United States	487	Baylor College of Medicine	70
2	Mainland China	353	Fondazione Telethon	69
3	Italy	128	University of Naples Federico II National	58
4	Japan	64	National Institutes of Health (NIH) United States	54
5	Canada	61	Harvard University	45
6	Germany	58	Institut National de la Sante et de la Recherche Medicale	38
7	France	57	University of California System	38
8	England	53	Johns Hopkins University	36
9	South Korea	52	University of Texas System	36
10	Spain	27	Chinese Academy of Sciences	30

A total of 1,340 institutes were participated in the TFEB research (Supplementary Figure 4). Table 3 lists the top 10 institutes in terms of publications. The 10 institutes account for 44.76% of total publications, among which Baylor College of Medicine has the largest publications, followed by Fondazione Telethon, University of Naples Federico II, National Institutes of Health (NIH) United States.

### Distribution of Authors

Over 6,842 authors contributed to TFEB research, the co-occurrence map of authors is shown in Supplementary Figure 5. Table 4 lists the top 10 authors in the number of publications. Ballabio A (62 publications) ranked first, followed by Argani P (22 publications), Chen Y (19 publications), and Li Y (19 publications).

**TABLE 4:** The top 10 authors, co-cited authors and co-cited references.

Rank	Author	Count	Co-cited author	Count	Co-cited references	Count
1	Ballabio A	62	Settembre C	663	Settembre et al., 2011, DOI 10.1126/science.120459	353
2	Argani P	22	Sardiello M	418	Settembre et al., 2012, DOI 10.1038/emboj.2012.32	300
3	Chen Y	19	Martina JA	298	Roczniak-Ferguson et al., 2012, DOI 10.1126/scisignal.2002790	237
4	Li Y	19	Roczniak-Ferguson A	271	Martina et al., 2012, DOI 10.4161/auto.19653	184
5	Liu Y	17	Medina DL	264	Medina et al., 2015, DOI 10.1038/ncb3114	180
6	Medina DL	17	Palmieri M	221	Settembre et al., 2013a, DOI 10.1038/ncb2718	179
7	Zhang L	17	Mizushima N	192	Sardiello et al., 2009, DOI 10.1126/science.1174447	174
8	Zhang Y	16	Napolitano G	160	Settembre et al., 2013b, DOI 10.1038/nrm3565	131
9	Li M	15	Klionsky DJ	158	DOI 10.1242/jcs.146365	129
10	Puertollano R	15	Decressac M	102	DOI 10.1093/hmg/ddr306	121

CiteSpace analyzed the information of author citations and visualized it in a co-citation network (Supplementary Figure 6). Among the top 10 co-cited authors (Table 4), Settembre C (663 co-citations) ranked first, followed by Sardiello M (418), Martina JA (298), Roczniak-Ferguson A (271), and Medina DL (264).

### Analysis of References

**FIGURE 3:**
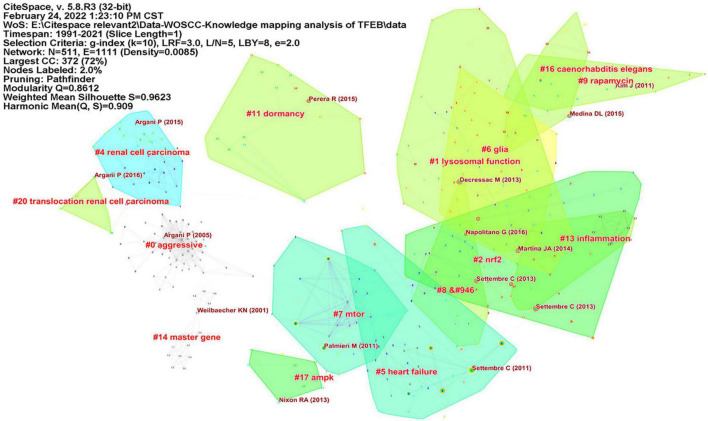
The reference co-citation clusters map for publications in TFEB research.

**FIGURE 4:**
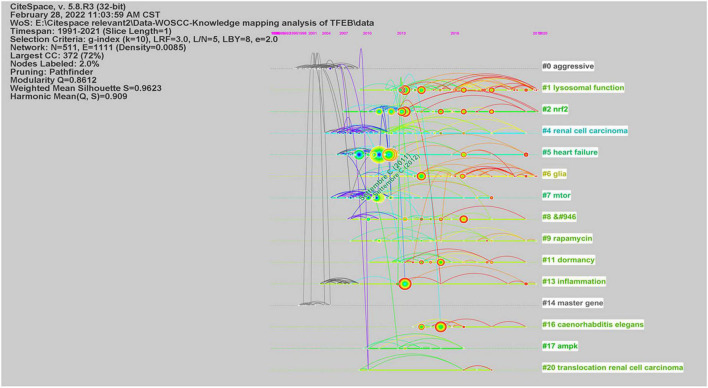
The timeline view of co-cited references from publications in TFEB research.

We used CiteSpace to analyze co-citation of references (Supplementary Figure 7), in Figure 3, network contained 511 nodes and 1111 links, the Modularity Q was 0.8612 (>0.5), meaning that the clusters of networks were reasonable, while the Mean Silhouette was 0.9623 (>0.5), indicating that the homogeneity of clusters were acceptable (Chen, 2006). In this network, more important clustering labels were listed in 21 clusters: #1 lysosomal function, #2 Nrf2, # 5 heart failure, # 6 glia, # 7 mTOR, # 9 rapamycin, # 11 dormancy, # 13 inflammation, # 16 caenorhabditis elegans, # 17 AMPK, # 20 translocation renal cell carcinoma. Furthermore, the timeline view of these clusters was shown in Figure 4.

### Analysis of Keywords

**FIGURE 5:**
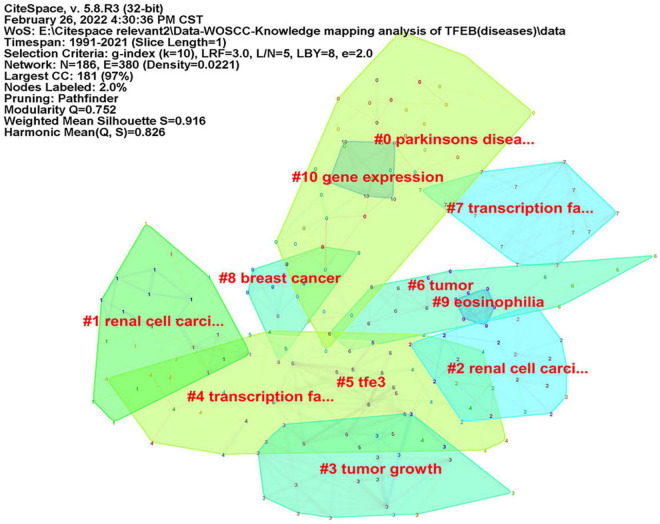
Keyword clusters map of TFEB about “diseases.”

**FIGURE 6:**
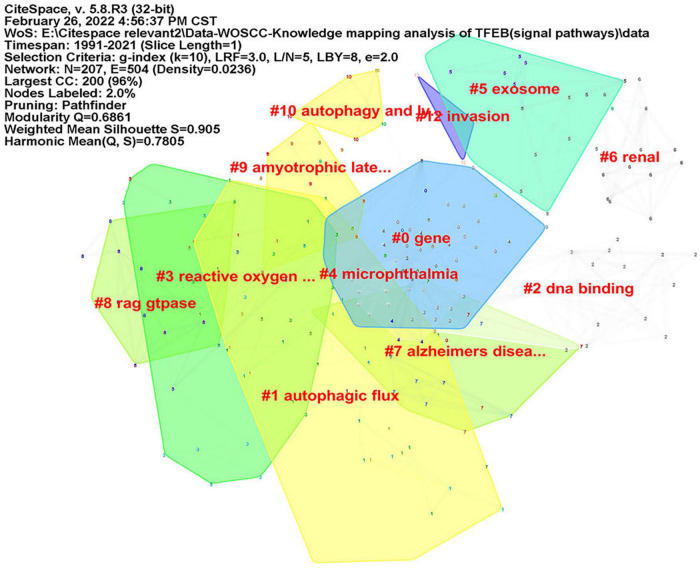
Keyword clusters map of TFEB about “signal pathways.”

**FIGURE 7:**
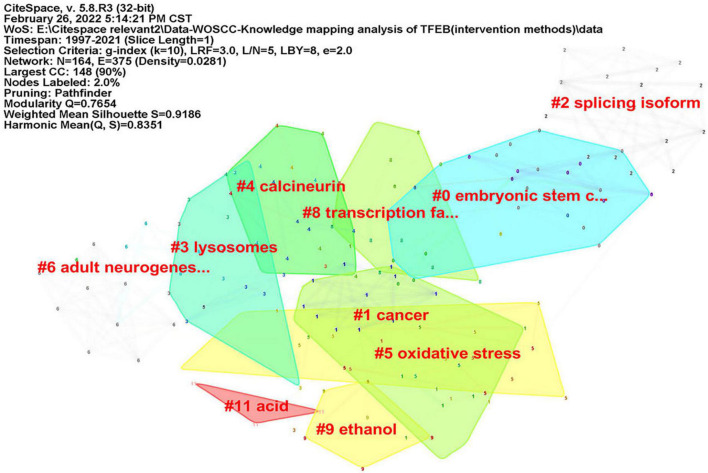
Keyword clusters map of TFEB about “intervention methods.”

CiteSpace was used to analyze keyword co-occurrence, in consideration of the different types of included literatures, two researchers (JZ) and (RZ) re-screened the 1,059 kinds of literatures according to three parts: “diseases,” “signal pathways,” and “intervention methods.” There were 402 articles concerning “diseases”, map of keyword co-occurrence resulted in 186 nodes and 380 links (Figure 5), the keywords were detected by Burst method and 19 words were obtained (Supplementary Figure 8). A total of 350 literatures were included concerning “signal pathways,” map of keyword co-occurrence resulted in 207 nodes and 504 links (Figure 6), the keywords were detected by Burst method and 21 words were obtained (Supplementary Figure 9). For “interventions,” 169 literatures were verified, map of keyword co-occurrence resulted in 164 nodes and 375 links (Figure 7), the keywords were detected by Burst method and 15 words were obtained (Supplementary Figure 10).

## Discussion

### General Information

The number of publications and citations has shown a gradual upward trend every year, indicating that TFEB research is still attracting attention. An article on TFEB was first published in 1991 (Fisher et al., 1991). From 1991 to 2021, the Research area mainly focused on Cell biology, Biochemistry Molecular biology and Oncology. As for published journals, the top three are *Autophagy*, *Nature Communication* and *Cell Death & Disease*, accounting for 12.65%. Among them, the impact factors of *Autophagy*, *Nature Communication*, *Proceedings of the National Academy of Sciences of the United States of America* and *Embo Journal* were more than 10 points, and show an increasing trend every year. Among the top 10 contributive countries/regions in TFEB research, The United States and Mainland China occupied the leading position, accounting for 79.32%. While from the distribution of institutes, the top 5 institutes were Baylor College of Medicine (United States), Fondazione Telethon (Italy), University of Naples Federico II (Italy), National Institutes of Health (NIH) (United States), Harvard University (United States), all of which were from the United States and Italy. Although the number of publications in mainland China was large, the research institutes were relatively scattered. In terms of the distribution of authors, Ballabio A ranked first in 58 publications. According to Price’s Law (from PRICE⋅D), the minimum number of publications for core authors N = 0.749$\sqrt{M\,\max}$ (*M*max is the publications of the most prolific authors), Calculated N≈6. In terms of the number of articles published, 53 authors have published more than 6, accounting for 6.96% (<50%), indicating that the core author team in this research field has not yet been formed.

### Citation Information

In Table 4, top 10 co-cited references are listed. Sardiello M published an article in Science in 2009 and found that most lysosomal genes exhibited coordinated transcriptional behaviors and were regulated by TFEB (Sardiello et al., 2009). Also published in *Science*, Settembre C demonstrated that TFEB was the main gene for lysosomal biogenesis, nuclear localization and activity of TFEB were regulated by serine phosphorylation mediated by the extracellular signal-regulated kinase 2 (ERK2) in 2011 (Settembre et al., 2011). In the following two years, researches showed that TFEB colocalized with master growth regulator mTOR complex 1 (mTORC1) on the lysosomal membrane, pharmacological inhibition of mTORC1, as well as starvation and lysosomal disruption, activated TFEB by promoting its nuclear translocation (Martina et al., 2012; Roczniak-Ferguson et al., 2012; Settembre et al., 2012). Settembre C clarified that TFEB was induced by starvation through an autoregulatory feedback loop and exerts a global transcriptional control on lipid catabolism via Ppargc1α and Ppar1α (Settembre et al., 2013a). Furthermore, TFEB also mediated lysosomal involvement in secretion, plasma membrane repair, signaling and energy metabolism. Targeting TFEB may provide a novel therapeutic strategy for modulating lysosomal function in human disease (Settembre et al., 2013b).

### Research Frontiers

The burst of keywords provided a reasonable prediction of the research front (Supplementary Figures 6, 8, 10). The beginning to the end of each burst interval was indicated by a red line. Here, we list three types of frontiers about TFEB research as follows:

For diseases: (1) NDs, more and more studies believe that the dysfunction of ALP leads to the failure of long-lived proteins to be degraded in a short time, and excessive accumulation leads to neurotoxicity. For example, Aβ deposition formed AD. Abnormal protein aggregation of mutant Cu/Zn superoxide dismutase 1 (SOD1) was the main pathological change of amyotrophic lateral sclerosis (ALS) (Beers and Appel, 2019). TFEB-mediated autophagy has a positive effect on improving this type of NDs (Chen et al., 2015; Song et al., 2021). (2) Tumors, renal-cell carcinoma (RCC) with t (6; 11) (p21; q12) was reported by Argani P in 2001 (Argani et al., 2001). The tumor is characterized by the fusion of the non-protein-coding gene Alpha (MALAT1) located at 11q12 and the TFEB gene of 6p21, which up-regulates the expression of TFEB, leading to abnormal expression of melanocyte markers (Argani et al., 2005; Rao et al., 2012), that is just the opposite of NDs in gene regulation. For melanoma, especially MiTF plays an important role in inducing autophagy (Ploper et al., 2015; Möller et al., 2019). It has been demonstrated that MiTF is an amplified oncogene in a fraction of human melanomas and that it also has an oncogenic role in human clear cell sarcoma (Levy et al., 2006). For pancreatic cancer, especially pancreatic ductal adenocarcinoma (PDA), researchers identified the MiT/TFE family of transcription factors as master regulators of metabolic reprogramming in PDA and demonstrate that transcriptional activation of clearance pathways converging on the lysosome is a novel hallmark of an aggressive malignancy (Perera et al., 2015). For breast cancer, significant TFEB high expression and lysosomal biosynthesis in early breast cancer defined poor prognosis (Giatromanolaki et al., 2017). Cell and animal experiments have suggested that TFEB is a major regulator of Tumor-associated macrophages (TAM) in breast cancer. TFEB controls TAM gene expression and function through multiple autophagy/lysosome-dependent and independent pathways. The efficacy of existing treatments, including breast cancer immunotherapy, could be improved in the future by modulating TAM function and tumor microenvironment (Li et al., 2020).

For signal pathways: (1) Rap-TRPML1-Calcineurin-TFEB, in the classic Rap-mTORC1-ULK1/TFEB signaling pathway, Rap could remove the inhibitory effect of mTOR on ULK1 to promote autophagy, and it also activated TFEB to up-regulate lysosomal function. The latest research shows that Rap can directly activate TRPML1 without mTOR (Li et al., 2021). CN acts as a sensor for calcium signals derived from this lysosome to activate TFEB, thereby enhancing lysosomal function and promoting autophagy (Medina and Ballabio, 2015; Zhang et al., 2019). (2) TFE3, additional to TFEB, TFE3 also regulate lysosomal autophagy in tissues (Martina et al., 2014, 2016; Pastore et al., 2016; Raben and Puertollano, 2016).

For interventions: (1) Trehalose, which is a non-reducing disaccharide, may become the best candidate for neurodegenerative diseases due to its non-toxicity. Trehalose induces autophagy through lysosomal mediated TFEB (Jeong et al., 2021), which can be used as a new drug choice for treating atherosclerosis (Evans et al., 2018), Motoneuron degeneration (Rusmini et al., 2019), cardiac remodeling (Sciarretta et al., 2018) and other diseases. (2) Curcumin, is identified as a novel mTOR-independent activator of TFEB (Song et al., 2016), which can directly bind to TFEB, promote TFEB nuclear translocation and increase transcriptional activity of TFEB (Zhang et al., 2016).

## Conclusion

This study will help researchers understand the trend of TFEB research. Disease research focuses more on NDs and tumors, specifically AD, PD, RCC, melanoma, breast cancer, and PDA. Trehalose and curcumin are novel agents acting on TFEB. Rap-TRPML1-Calcineurin-TFEB and TFE3 are increasing signal pathway researches, similarly, the molecular biological mechanism of TFEB needs further exploration.

## Data Availability Statement

The original contributions presented in the study are included in the article/Supplementary Material, further inquiries can be directed to the corresponding author.

## Ethics Statement

Ethical review and approval was not required for the study on human participants in accordance with the local legislation and institutional requirements. Written informed consent for participation was not required for this study in accordance with the national legislation and the institutional requirements.

## Author Contributions

RZ and XLL conceived and designed the experiments, performed the experiments, analyzed the data, contributed analysis tools, authored or reviewed drafts of the manuscript, and approved the final draft. DL performed the experiments, analyzed the data, prepared figures and/or tables, and approved the final draft. ZL and JZ performed the experiments, prepared figures and/or tables, and approved the final draft. XinL and XiaL conceived and designed the experiments. XiaL authored and approved the final draft.

## Conflict of Interest

The authors declare that the research was conducted in the absence of any commercial or financial relationships that could be construed as a potential conflict of interest.

## Publisher’s Note

All claims expressed in this article are solely those of the authors and do not necessarily represent those of their affiliated organizations, or those of the publisher, the editors and the reviewers. Any product that may be evaluated in this article, or claim that may be made by its manufacturer, is not guaranteed or endorsed by the publisher.

## Supplementary Material

Supplementary Figure 1 The number of Publications and citations in TFEB research.

Supplementary Figure 2 The dual-map overlay of journals related to TFEB research.

Supplementary Figure 3 The network map of countries/regions that involved inTFEB research.

Supplementary Figure 4 The network map of institutes that involved in TFEB research.

Supplementary Figure 5 The network map of active authors contributed to TFEB research.

Supplementary Figure 6 The co-citation map of authors contributed to TFEB research.

Supplementary Figure 7 The co-citation map of references from publications inTFEB research.

Supplementary Figure 8 Top 19 keywords with strongest citation bursts in thepart of “diseases.”

Supplementary Figure 9 Top 21 keywords with strongest citation bursts in thepart of “signal pathways.”

Supplementary Figure 10 Top 15 keywords with strongest citation bursts in thepart of “intervention methods.”
