# Supplementary figures and images for: Research Hotspots and Trends Analysis of TFEB: A Bibliometric and Scientometric Analysis
Source: Front Mol Neurosci. 2022 Apr 21;15:854954. doi: 10.3389/fnmol.2022.854954 (PMC9069162; doi:10.3389/fnmol.2022.854954)

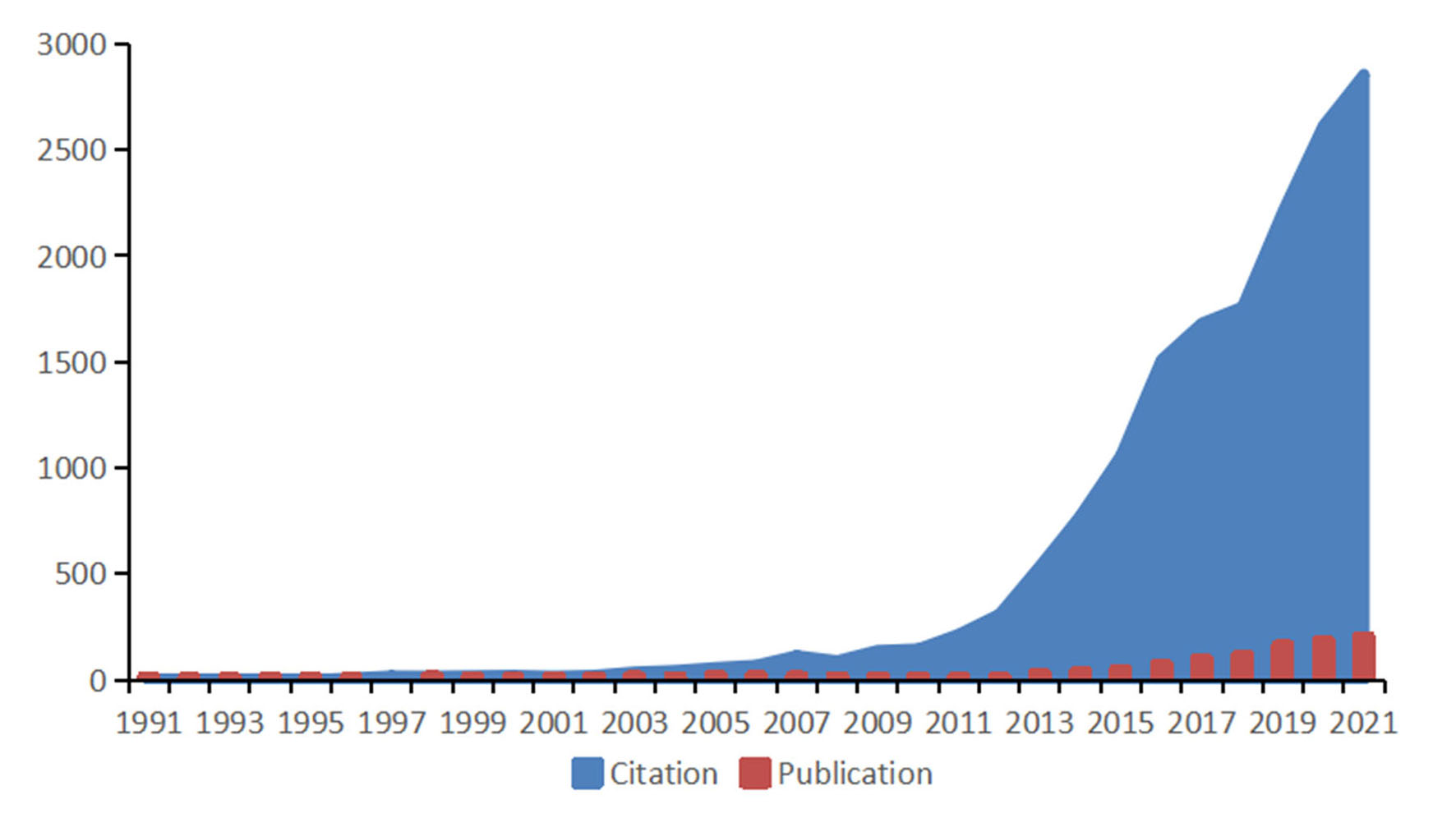

Supplement: Supplementary Figure 1 — The number of Publications and citations in TFEB research. [file Image_1.JPEG]

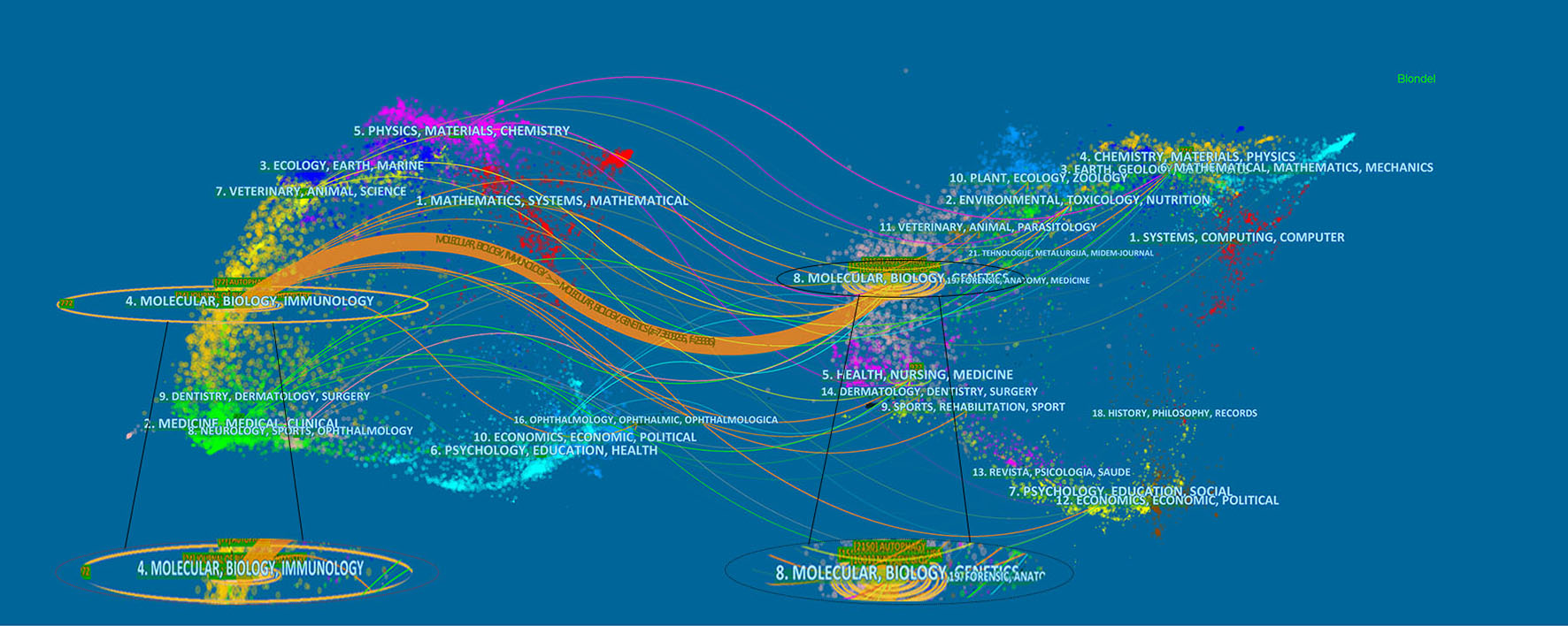

Supplement: Supplementary Figure 2 — The dual-map overlay of journals related to TFEB research. [file Image_2.JPEG]

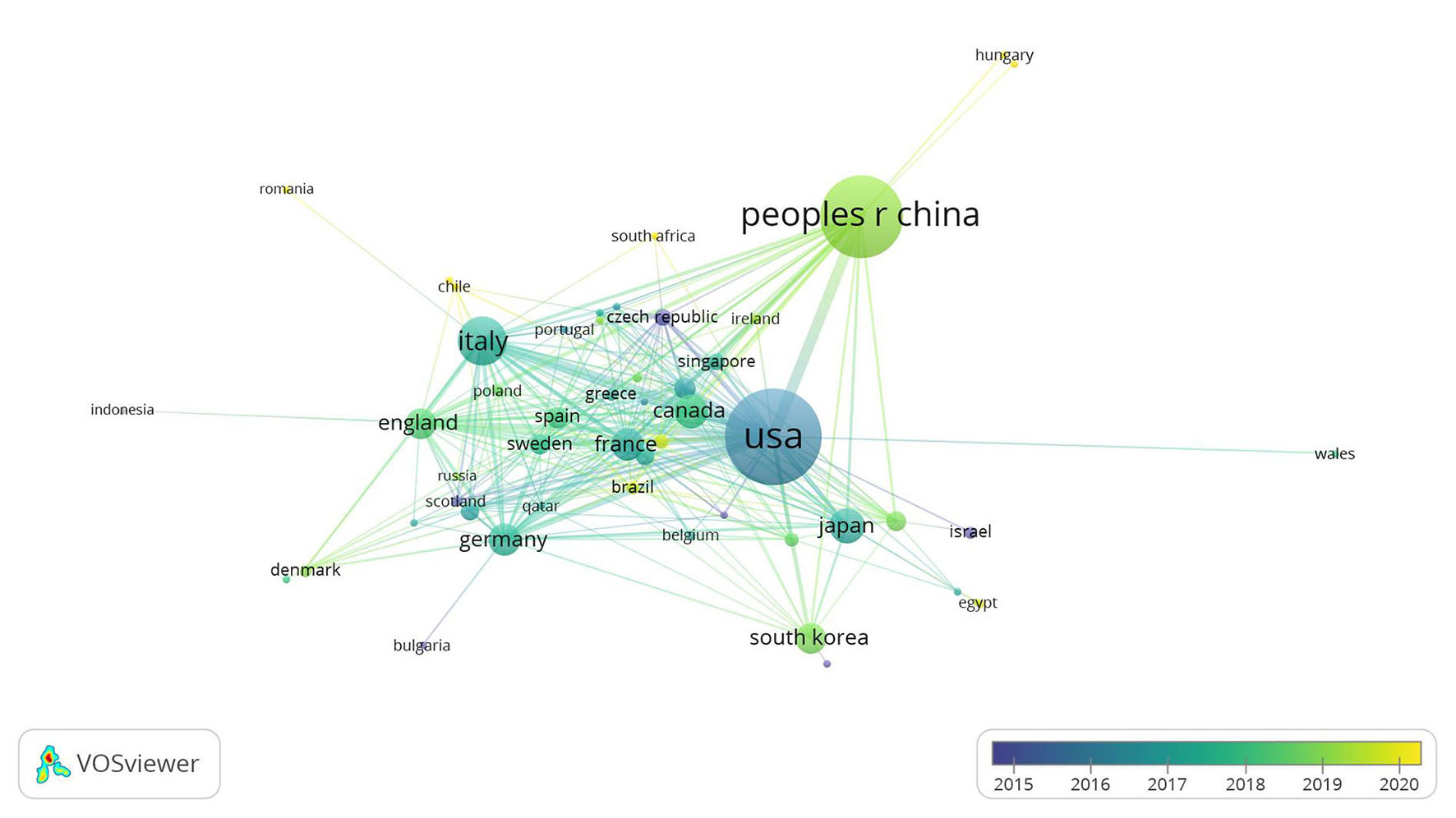

Supplement: Supplementary Figure 3 — The network map of countries/regions that involved inTFEB research. [file Image_3.JPEG]

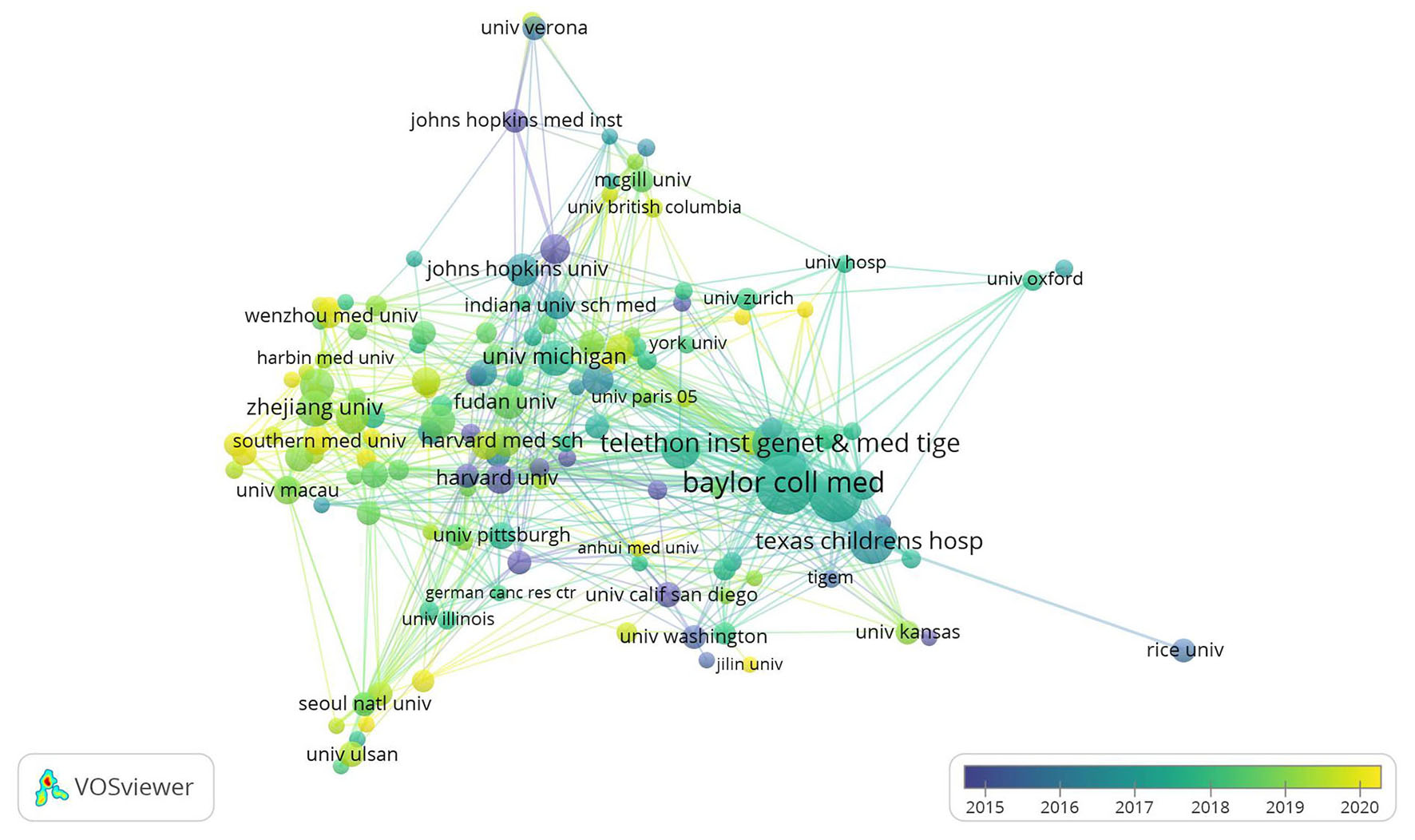

Supplement: Supplementary Figure 4 — The network map of institutes that involved in TFEB research. [file Image_4.JPEG]

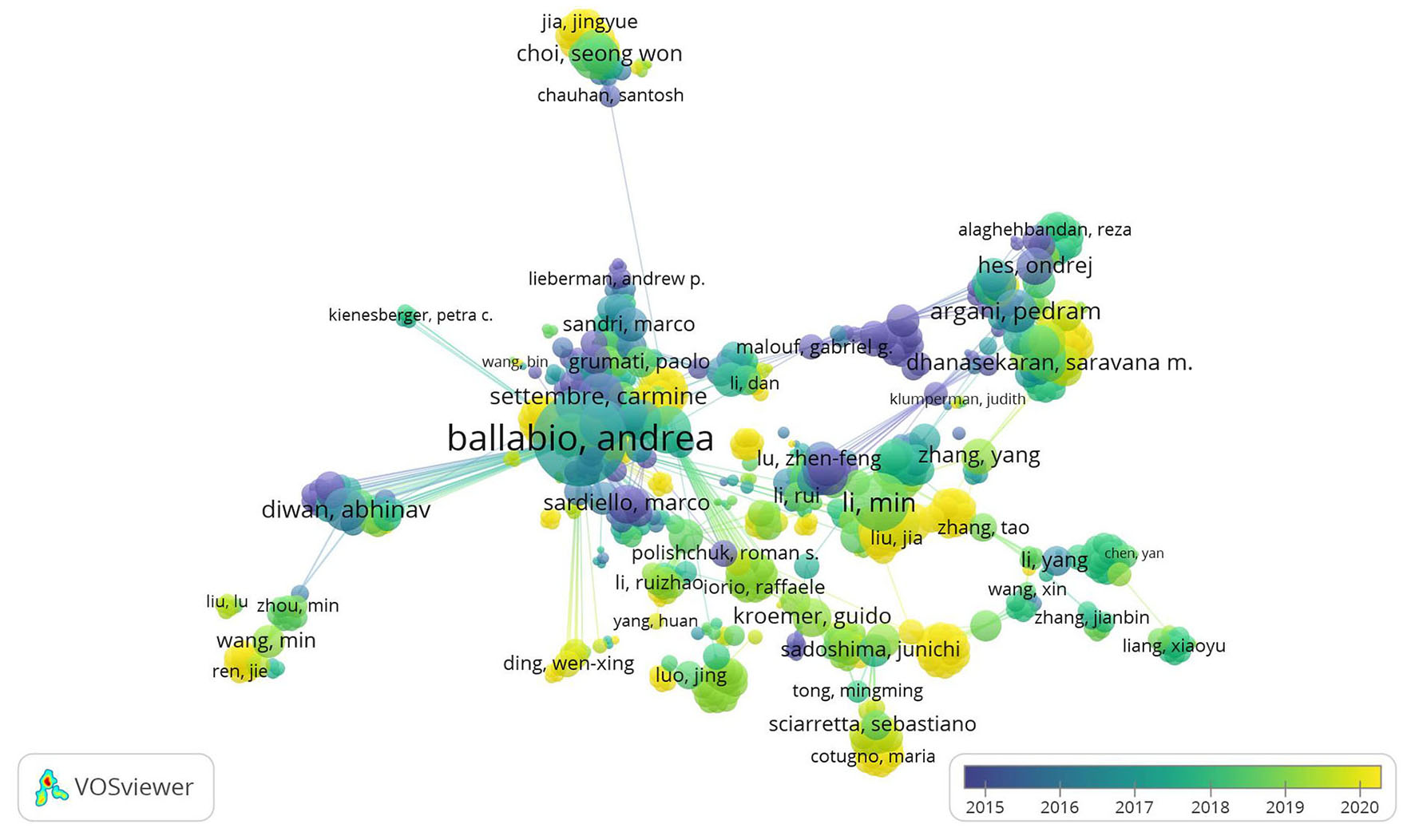

Supplement: Supplementary Figure 5 — The network map of active authors contributed to TFEB research. [file Image_5.JPEG]

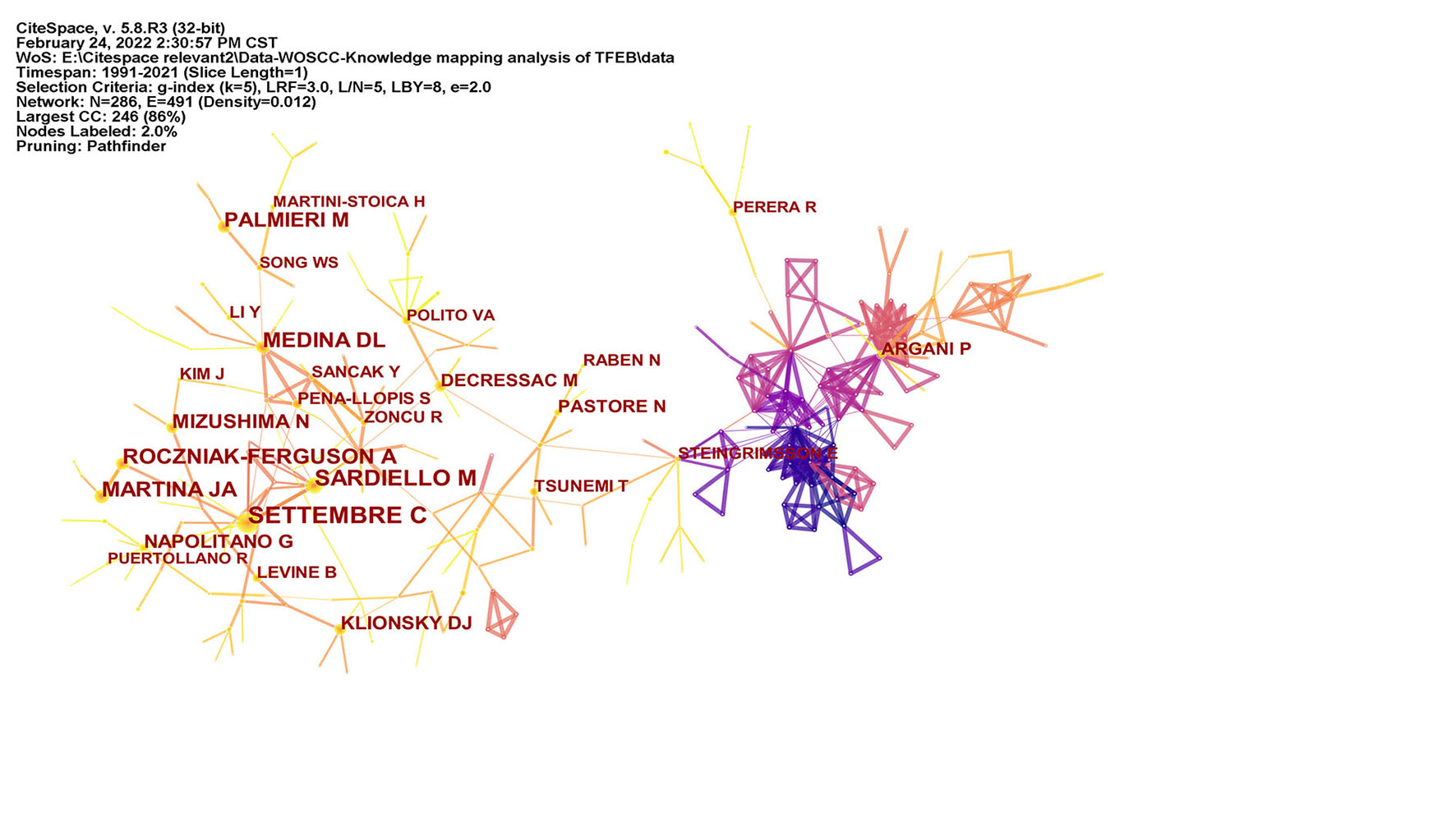

Supplement: Supplementary Figure 6 — The co-citation map of authors contributed to TFEB research. [file Image_6.JPEG]

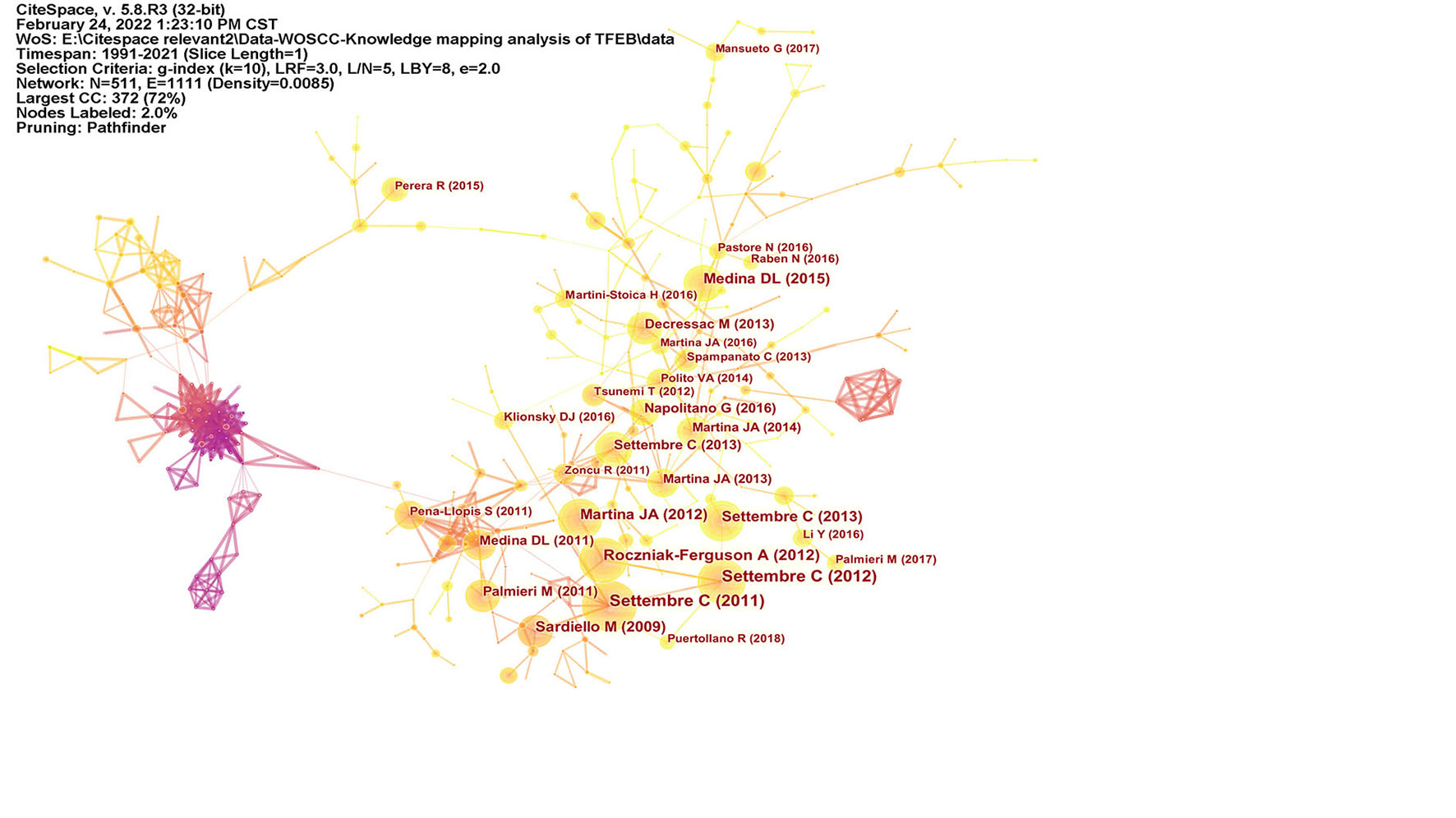

Supplement: Supplementary Figure 7 — The co-citation map of references from publications inTFEB research. [file Image_7.JPEG]

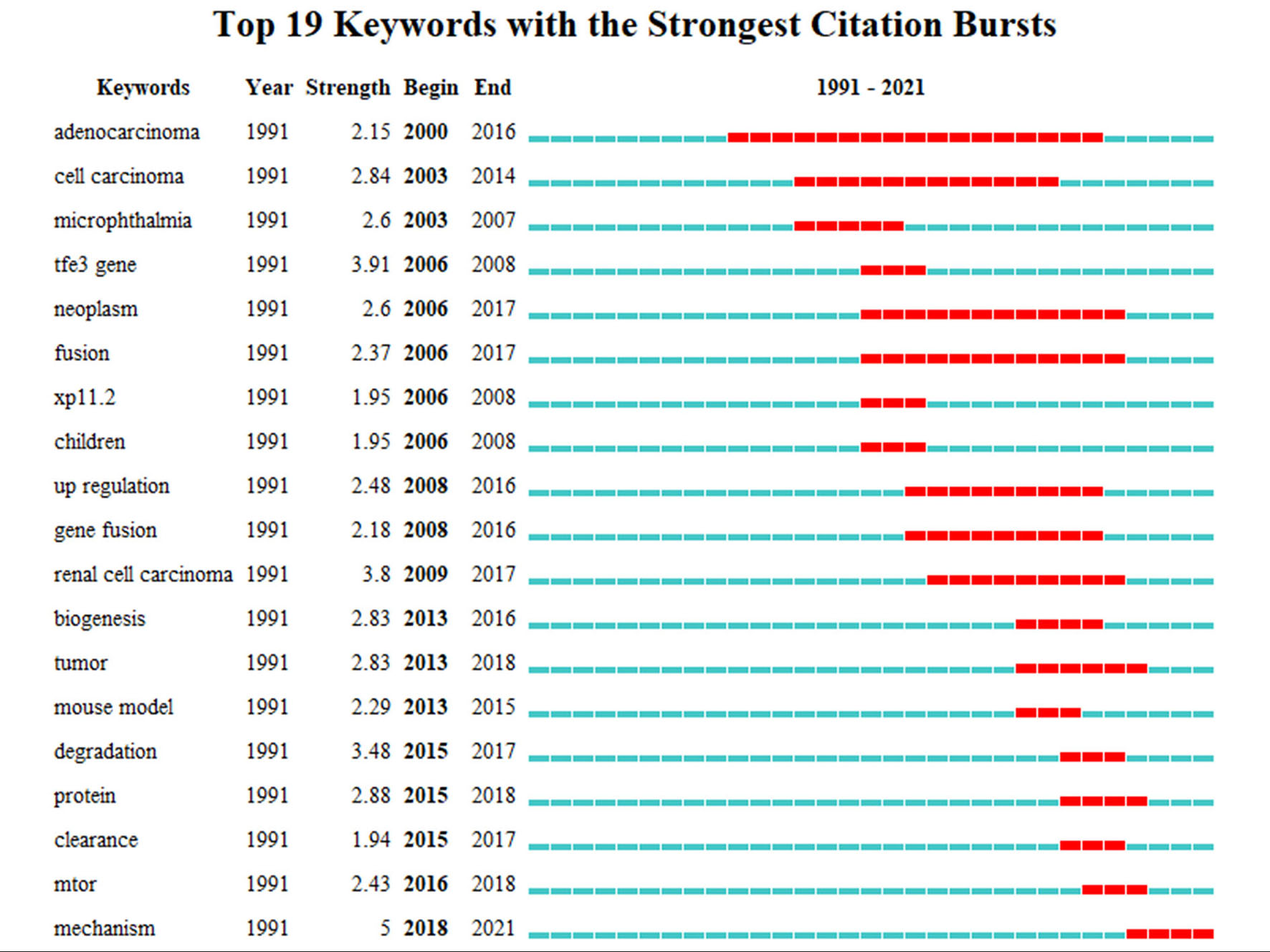

Supplement: Supplementary Figure 8 — Top 19 keywords with strongest citation bursts in thepart of “diseases.” [file Image_8.JPEG]

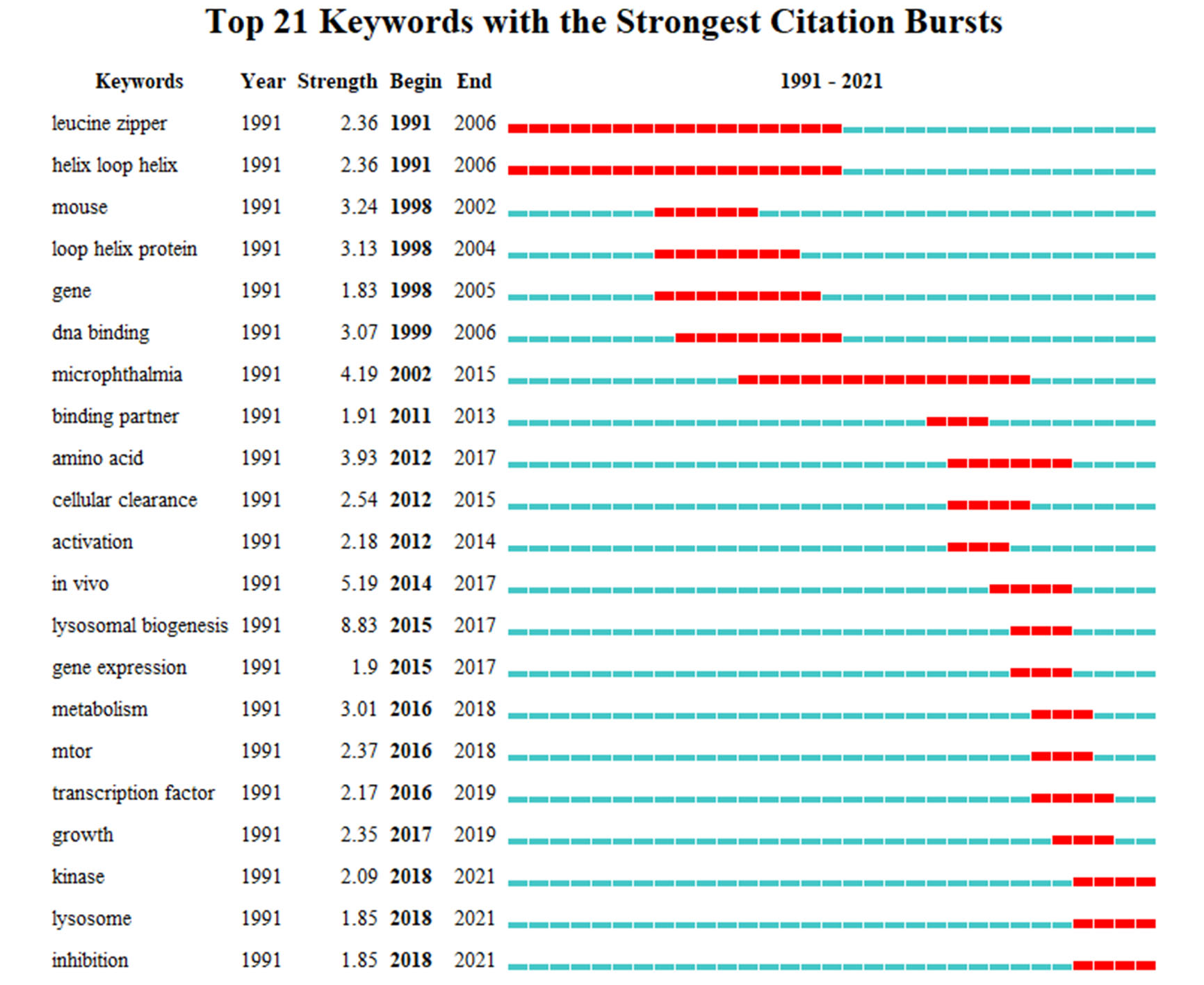

Supplement: Supplementary Figure 9 — Top 21 keywords with strongest citation bursts in thepart of “signal pathways.” [file Image_9.JPEG]

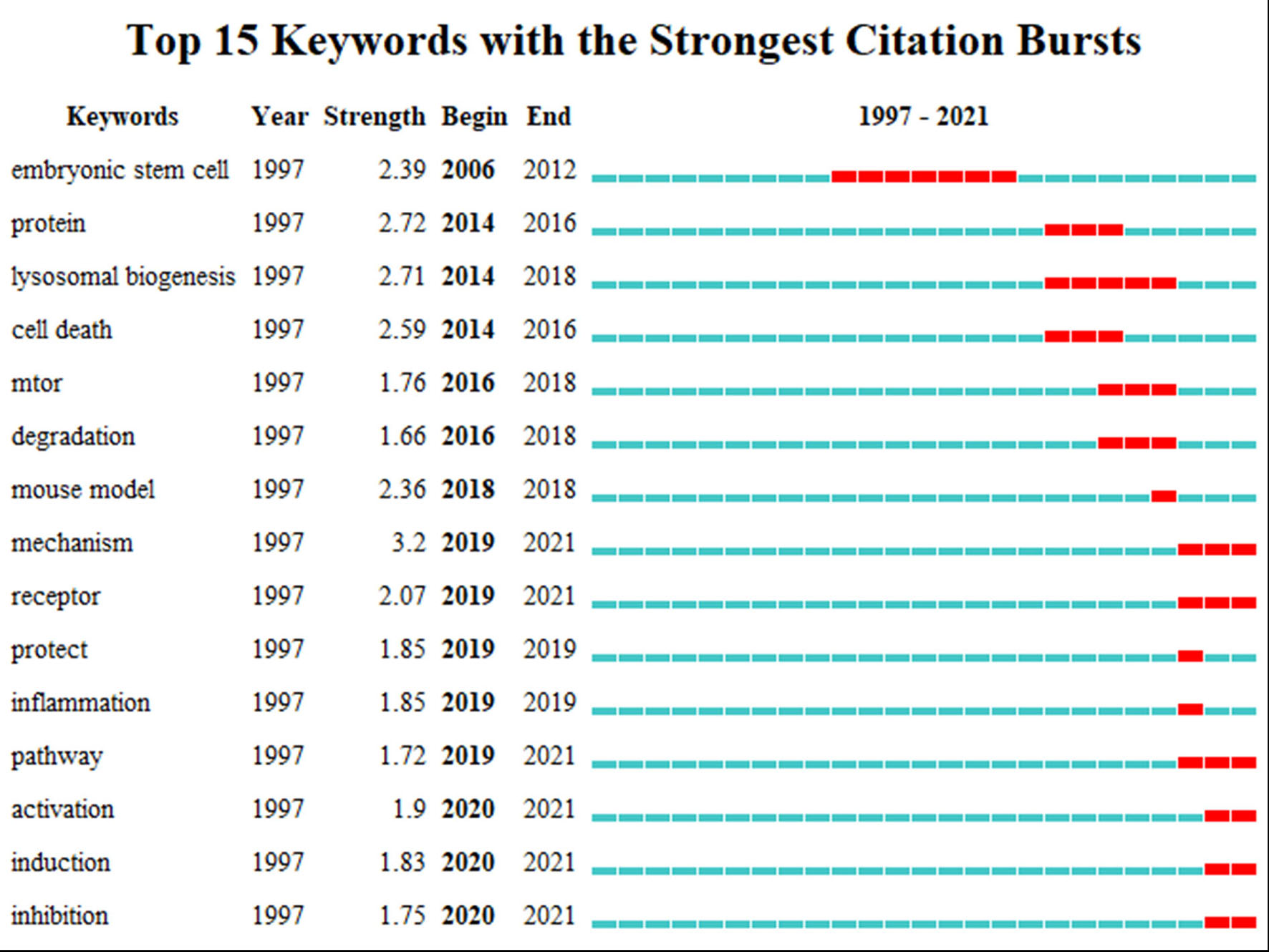

Supplement: Supplementary Figure 10 — Top 15 keywords with strongest citation bursts in thepart of “intervention methods.” [file Image_10.JPEG]
